# Visual Perception Supports Adults in Numerosity Processing and Arithmetical Performance

**DOI:** 10.3389/fpsyg.2021.722261

**Published:** 2021-10-22

**Authors:** Xinyao He, Xinlin Zhou, Jin Zhao, Yiyun Zhang

**Affiliations:** ^1^School of Psychology, Liaoning Normal University, Liaoning, China; ^2^State Key Laboratory of Cognitive Neuroscience and Learning, Siegler Center for Innovative Learning, Advanced Innovation Center for Future Education, Beijing Normal University, Beijing, China; ^3^Dalian Institute of Science and Technology, Liaoning, China

**Keywords:** visual perception, numerosity processing, arithmetical performance, adults, perceptual processing

## Abstract

Previous studies have found a correlation between numerosity processing and arithmetical performance. Visual perception has already been indicated as the shared cognitive mechanism between these two; however, these studies mostly focused on children. It is not clear whether the association between numerosity processing and arithmetical performance still existed following the development of individual arithmetical performance. Consequently, the underlying role of visual perception in numerosity processing and arithmetical performance has not been sufficiently studied in adults. For this study, researchers selected a total of 205 adult participants with an average age of 22years. The adults were administered arithmetic tests, numerosity comparison, and visual figure matching. Mental rotation, choice reaction time, and nonverbal intelligence were used as cognitive covariates. Results showed that numerosity comparison of adults correlated with their arithmetical performance, even after controlling for age and gender differences as well as general cognitive processing. However, after controlled for visual figure matching, the well-established association between numerosity comparison and arithmetic performance disappeared. These results supported the visual perception hypothesis, that visual perception measured by visual figure matching can account for the correlation between numerosity comparison and arithmetic performance. This indicated that even for adult populations, visual perceptual ability was the underlying component of numerosity processing and arithmetic performance.

## Introduction

Previous studies have shown that numerosity processing (e.g., comparison of numbers of dots in two arrays) is associated with children’s mathematical performance (e.g., Halberda et al., 2008; Mussolin et al., 2012; Lonnemann et al., 2013; Keller and Libertus, 2015; Matthews et al., 2016; Zhang et al., 2016). Some studies indicate that this connection may be due to the fact that both of these share magnitude processing or approximate numerical estimation (e.g., Halberda et al., 2008; Libertus et al., 2011; Lyons and Beilock, 2011). Zhou et al. (2015) have put forward the visual perception hypothesis to support this association. Visual perception was found to contribute to both numerosity processing (e.g., Gebuis and Reynvoet, 2012; Gebuis et al., 2014; Leibovich-Raveh et al., 2017) and mathematical performance (e.g., Kurdek and Sinclair, 2001; Fuchs et al., 2006, 2010; Berg, 2008; Wei et al., 2012b). In addition, some studies have shown that visual perception can explain the association between numerosity processing and mathematical performance (e.g., Zhou et al., 2015; Zhou and Cheng, 2015; Wang et al., 2016; Cui et al., 2017; Zhang et al., 2019).

Like previous research, these studies focused on children, and researchers did not know whether the role of visual perception in numerosity processing and mathematical performance varied with development, especially in adults. In the current study, researchers examine the role of visual perception in numerosity processing and mathematical performance of adults, in order to test the developmental stability of visual perception hypothesis.

### Numerosity Processing and Mathematical Performance

A lot of studies have shown a correlation between numerosity processing and mathematical performance, but most of them focused on children (e.g., Halberda et al., 2008, 2012; Mundy and Gilmore, 2009; Inglis et al., 2011; Libertus et al., 2011, 2013; Bonny and Lourenco, 2013). The first line of supported references comes from the research on children with dyscalculia, which show the importance of numerosity processing in arithmetical performance (e.g., Landerl et al., 2004; Butterworth, 2005; Iuculano et al., 2008). For example, Piazza et al. (2010) found that the numerosity comparison ability of 10-year-old children with dyscalculia was significantly lower than that of normal children. And their score on numerosity processing task was only equal to that of normal 5-year-old children.

Individual differences in numerosity processing also correlated with mathematical performance for normally developing children (e.g., Halberda et al., 2008, 2012; Mundy and Gilmore, 2009; Inglis et al., 2011; Libertus et al., 2011, 2013; Bonny and Lourenco, 2013). Halberda et al. (2008) found that 14-year-old children’s performance on the numerosity comparison task correlated with scores on standardized mathematics achievement tests. Training studies based on numerosity processing also supported the correlation between numerosity processing and individual mathematical performance (Obersteiner et al., 2013; Park and Brannon, 2013).

Different from many studies on children, there are a few of studies exploring the association between numerosity processing and mathematical performance on adults. They found that the numerosity comparison of adults was significantly correlated with their mathematical performance (Guillaume et al., 2013; Szucs et al., 2013; Haist et al., 2015; Dietrich et al., 2017), even after controlling for general cognitive processes including working memory, and rapid automatized naming (Mazzocco et al., 2011; Halberda et al., 2012; Libertus et al., 2012). For example, Mazzocco et al. (2011) found the correlation between numerosity comparison and adults’ performance on mathematical performance measured by two standardized mathematics tests, the Test of Early Mathematics Ability–Second Edition (TEMA–2; Ginsburg and Baroody, 1990), and the Woodcock–Johnson Revised Calculation subtest (WJR–Calc; Woodcock and Johnson, 1989) which involved formal skills such as counting and reading and writing numerals, and informal skills such as cardinality. The correlation still existed even after controlling for general cognitive factors measured by rapid automatized naming-color, rapid automatized naming-number, digits forward memory, and digits backward memory. Similar to that, Dietrich et al. (2017) also proved the correlation between numerosity processing and arithmetical performance for adults. In particular, they also found that visual strategy was the most commonly used strategy for participants when solving the numerosity comparison and arithmetical tasks. Haist et al. (2015) conducted a developmental fMRI study focused on individual performance on numerosity comparison task. They found that the numerosity comparison relied more on the ventral occipital–temporal cortex and hippocampus, and the activation in these cortexes significantly correlated with adults’ behavior performance in mathematical achievement measured by the Woodcock-Johnson III.

The correlation between numerosity processing and mathematical performance has been traditionally attributed to the number-specific processing in these tasks (Halberda et al., 2008), since both numerosity processing and symbolic numerical skills involve quantity processing. Previous studies have shown that numerosity processing is important for the acquisition of symbolic numerical skills, such as counting and arithmetic (Gilmore et al., 2007; Inglis et al., 2011). Previous correlational studies have shown a significant relation between symbolic mathematical performance and quantity processing (e.g., Smedt et al., 2009; Sasanguie et al., 2012b; Zhang et al., 2016).

In contrast, some studies have questioned the magnitude hypothesis, showing conflicting results of the association between numerosity processing and mathematical performance for both children (e.g., Holloway and Ansari, 2009; Soltész et al., 2010; Sasanguie et al., 2012a, 2014; Vanbinst et al., 2012; Fuhs and Mcneil, 2013), and adults (Inglis et al., 2011; Lyons and Beilock, 2011; Price et al., 2012). For example, Sasanguie et al. (2012a) conducted a study on kindergarten students of grade one, grade two, and grade six. They did not find a significant correlation between number processing and mathematical performance measured by curriculum standardized mathematics achievement test. The test included 60 items, involving number knowledge, operation understanding, arithmetic, problem solving, measurement, and geometry. Vanbinst et al. (2012) also found no relation between numerosity processing and general mathematics achievement, defined by word problem-solving, multi-digit calculation, and geometry. Inglis et al. (2011) showed the correlation between numerosity processing and mathematical performance on children but not on adults. That might come from the diverse mathematical abilities involved in the mathematical tests, which including the calculation, math fluency, applied problems, quantitative concepts, and number series.

Apart from this, numerosity processing correlated with mathematical processing along with mathematical fluency, such as arithmetic fluency and symbolic numerical comparison (e.g., Rousselle and Noël, 2007; Smedt et al., 2009). It was not associated with others that have slow and complex processes, such as approximate computation and mathematical reasoning (e.g., Holzman et al., 1982, 1983; Jia et al., 2011; Wang et al., 2016; Cui et al., 2017; Zhang et al., 2019). For example, Zhang et al. (2016) investigated whether numerical processing played an important role in two types of mathematical competence: arithmetical computation and mathematical reasoning. The results showed that both non-symbolic numerical processing (numerosity comparison) and symbolic numerical processing (digit comparison) could independently predict arithmetic computation. Moreover, neither could predict mathematical reasoning.

### Visual Perception Hypothesis for the Association Between Numerosity Processing and Mathematical Performance

Recently, Zhou et al. (2015) proposed the visual perception hypothesis for the association between numerosity processing and mathematical performance. They thought that visual form perception accounted for the close relation between the numerosity processing and mathematical performance. According to the domain-general visual perception hypothesis, visual perception is the underlying processing of numerosity processing and mathematical performance. During the processing stage, both of them relied on rapid processing of visual Arabic symbols and signs for mathematics, like Arabic numbers, operational signs, and vertices of dot arrays that are used in numerosity processing. Previous studies have further examined the important role of visual perception in numerosity processing and mathematical performance with several lines (e.g., Zhou et al., 2015, 2020; Zhou and Cheng, 2015; Wang et al., 2016; Cui et al., 2017).

First, individual visual perception was found to correlate with mathematical performance in normally developing children (e.g., Zhou et al., 2015; Zhou and Cheng, 2015; Wang et al., 2016; Cui et al., 2017) and children with mathematical disabilities (e.g., Landerl et al., 2004; Geary et al., 2009; Piazza et al., 2010). Rosner (1973) measured visual perception with a visual perception test called VAT (Rosner, 1969), in which children were asked to match a target stimulus by copying designs drawn on matrices of dots. The result showed that visual perception could predict the changes of computational performance after controlling for auditory perception. Sigmundsson et al. (2010) studied children with low mathematics performance and found that children’s sensitivity to visual coherence was lower than that of the control group of the same age. Based on this study, Boets et al. (2011) further proved that the sensitivity of visual coherent motion is a predictor of simple subtraction.

Second, numerosity is also defined by its visual characteristics (Clearfield and Mix, 2001; Durgin, 2008; Dakin et al., 2011; Gebuis and Gevers, 2011; Gebuis and Reynvoet, 2012; Gebuis et al., 2014; Morgan et al., 2014; Leibovich-Raveh et al., 2017). According to Gebuis and Reynvoet (2012), a numerosity comparison task was influenced by five visual properties: envelope area or convex hull, circumstance, item size, density, and total surface area. Density is the envelope area divided by total surface. Previous studies have found that numerosity discrimination relies highly on visual properties, including the convex hull (Gebuis and Gevers, 2011), total item perimeter (Clearfield and Mix, 2001), density (Durgin, 2008), the contrast energy at high spatial frequencies (Dakin et al., 2011; Morgan et al., 2014), and total item surface area (Feigenson et al., 2002). Therefore, when the number of objects included in a numerosity task changes, other visual properties must change accordingly.

Third, patients who suffer from visual form agnosia had difficulties in the numbers and mathematical signs (Milner et al., 1991; Cavina-Pratesi et al., 2015). For example, patient DF suffers from a permanent visual form agnosia, so she cannot distinguish single alphanumeric characters or simple geometric shapes. This was attributed to the loss of the bilateral lateral occipital areas (Milner et al., 1991). When only one point is displayed, the visual digital performance of DF has no problem. But following the increase in the number of points, the visual digital performance of DF decreased rapidly, the accuracy of two points is 33%, and the accuracy of three to five points is 0%. This is not due to her ability to count, because she can correctly count to six auditory taps. The impairment of visual number ability may be due to the defect of visual form perception.

In addition, the visual perception has also been found to account for the association between numerosity processing and mathematical performance, especially for arithmetical performance (e.g., Zhou et al., 2015; Zhou and Cheng, 2015; Wang et al., 2016; Cui et al., 2017, 2019; Zhang et al., 2019). Visual perception has been correlated with both numerosity processing and arithmetic fluency, even after controlling for the general cognitive processing (Zhou et al., 2015; Wang et al., 2016; Cui et al., 2017). The correlation between numerosity processing and arithmetic fluency was no longer significant after controlling for visual perception scores (Zhou et al., 2015; Cui et al., 2017; Zhang et al., 2019).

Cui et al. (2019) found that both reading comprehension and arithmetic fluency relied on visual perception, and the correlation with numerosity processing was fully accounted for by visual perception. Additionally, when compared with normally developing children, children with dyscalculia showed poorer performance in both numerosity processing and visual perception. But after controlling for visual form perception, the differences in numerosity processing between the two groups disappeared (Zhou and Cheng, 2015). Furthermore, studies have shown that short-term numerosity training enhances the arithmetical performance of children with dyscalculia by improving their visual perceptual performance (Cheng et al., 2019).

### Aim and Hypothesis

The aim of the current study was to test the visual perception hypothesis for the relationship between numerosity processing and mathematical performance in adult populations. There has been previous evidence to show the important role of visual perception in numerosity processing and mathematical performance; however, no studies explored the development effects on the association. The current investigation would fill the gap by examining the role of visual perception in numerosity processing and mathematical performance for adults. The general cognitive factors including spatial processing, general intelligence, processing speed were used as covariates. All of them are important cognitive factors for arithmetical performance (e.g., Swanson and Sachse-Lee, 2001; Passolunghi and Pazzaglia, 2004). Previous studies on the association between numerosity processing and mathematical performance also used the above cognitive factors as control variables on children (e.g., Zhou et al., 2015; Zhang et al., 2016, 2019; Cui et al., 2017).

In this study, we first tested whether numerosity processing still correlated with arithmetical performance for adults, as previous studies indicated (e.g., Mazzocco et al., 2011; Guillaume et al., 2013; Szucs et al., 2013). To extend the previous work, we tested adults about the role that visual perception played in the relationship between numerosity comparisons and arithmetic performance. There is still a question of addressing the developmental cognitive mechanism for the association between numerosity processing and arithmetic computation.

The hypotheses of the current study are that visual perception hypothesis is stable for adults, in particular, the association between numerosity processing measured by numerosity comparison and arithmetical performance measured by simple and complex subtraction still existed for adults in the current study. More important, visual perception can account for the association between them, even after controlling for age, gender differences and general cognitive processes.

## Materials And Methods

### Participants

A number of 205 healthy, right-handed university students, composed of 111 males and 94 females, were recruited from Beijing Normal University in China. The average age of the participants was 22.1years, ranging from 16.1 to 29.1years. They self-reported having normal or corrected-to-normal eyesight and normal hearing. Informed written consent was obtained from each participant after procedures were fully explained. Participants were given informed consent before the experiment and were debriefed with the research purpose after the experiment. The study received ethical approval from the Ethics Committee of the Faculty of Education at Beijing Normal University.

### Tests

**Figure 1:**
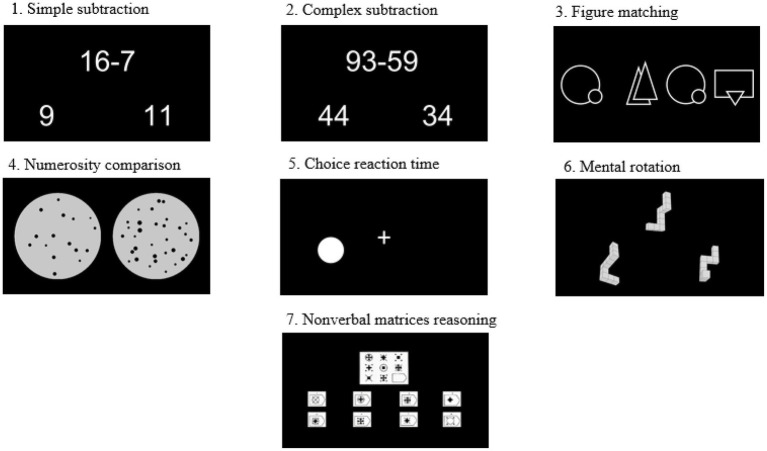
Example of stimuli for all tests.

Seven tests were administered using a web-based psychological testing system,^1^ including simple subtraction, complex subtraction, figure matching, numerosity comparison, choice reaction time, nonverbal matrices reasoning, and mental rotation (see Figure 1). Each test is divided into practice and formal tests. There are four to six trials in practice session which were using the same procedure as the formal test. Responses in all of the tests involved two choices and the correct answer was balanced across the two alternatives.

#### Arithmetic Computation

##### Simple Subtraction

The simple subtraction task was the reverse operation of a one-digit addition, with a range of minuends from 2 to 18 and a range of subtractions from 1 to 9. There were total 92 trials. The answer was determined to be a one-digit number. The incorrect candidate answers were the correct answer plus or minus 1, 2, or 3. Participants were asked to press “Q” with their left forefinger if the answer on the left was correct or “P” with their right forefinger if the answer on the right was correct. This task was limited to 2min.

##### Complex Subtraction

The complex subtraction calculation included 95 trials. The minuend and the subtraction were both two-digit, and most of the trials need to be borrowed. Each answer has two choices, 1 or 10 apart. This task was also limited to 2min.

#### Figure Matching

As in previous studies, we used figure matching tests to measure rapid visual perception ability (Zhou et al., 2015; Zhou and Cheng, 2015). The test included 120 trials, divided into three sessions with 40 trials in each session. In each trial, one figure was presented on the left side, and three figures were presented on the right side at the same time. Participants were required to answer whether the right figures included the left one. The “Q” key was for yes and the “P” key was for no. All trials were constructed from 150 abstract figures and presented for 400ms. Participants were asked to complete all of the trials.

#### Numerosity Comparison

Numerosity processing was measured by numerosity comparison. This test included 120 trials divided into three sessions. In each trial, two arrays of dots appeared on the screen at the same time. Participants were asked to judge which array contained more dots and pressed “Q” for the left choice and “P” for the right choice. The number of dots in each trial changed from 5 to 32, and the ratio of dots between the two dot arrays was between 1.14 and 2. Each trial was presented for 200ms.

The dots arrays were constructed by three conditions. First, the total combined area and average area of all dots in the dot array were systematically changed. Half of the trials in each proportion were point-size controlled, which means that the average area of the dots occurring in each trial was the same. In these trials, a lattice with more dots necessarily occupied a larger screen area. In the other half of the trials, the two arrays had the same combined area, meaning that the dots occupied the same total cumulative area in both arrays. In these types of trials, the more dots the array had the smaller the average point. The construction of dots in this study was based on the research of Halberda et al. (2008). The second rule was that the diameters of points in each dot array are pseudo-random, and the constraint was that the total area or average area of all dots in the dot array and the other dot arrays is equal. Third, the dots are randomly distributed within a circle.

#### Choice Reaction Time

A choice reaction time (CRT) task was used to control for the effect of processing speed. This test included 30 trials. In each trial, there was a fixation cross with a white dot on its left or right presented on the screen. The participants were asked to press “Q” with their left forefinger if the dot appeared on the left side of the fixation cross or “P” with their right forefinger if it appeared on the right side of the fixation cross. The position where the stimulus occurred on the screen was within 15° of visual angles. The stimulation intervals were random, ranging from 1,500 to 3,000ms. Participants’ keypress started the next trial.

#### Mental Rotation

This task was adapted from the mental rotation task used by Shepard and Metzler (1971). Mental rotation is different from passive spatial working memory, it is associated with active spatial working memory (Vecchi and Girelli, 1998). High scores in active spatial working memory indicate that participants do not simply passively memorize, but actively manipulate spatial information. In this test, one three-dimensional image appeared at the top of the screen and another two on the bottom. Participants were asked to determine whether the three-dimensional image at the top can be mentally rotated to match one of the other two images at the bottom of the screen. Participants were asked to respond by pressing the “Q” or “P” key on their keyboard. The test included 180 trials, and the time limit was 3min.

#### Nonverbal Matrices Reasoning

This test was adapted from the Raven’s Progressive Matrices test (Raven, 1998). It was used to control for the influence of general intelligence. For each trial, there was an incomplete figure presented on the top of the screen and two segments on the bottom. Participants were asked to judge which segment on the lower part of the screen could complete the incomplete figure’s inherently regular pattern, by pressing the “Q” button to choose the segment on the left-bottom or the “P” button to choose the segment on the right-bottom. The test time was limited to 4min. According to the previous research, there were total 80 trials including 44 projects selected from the standard progressive matrix and 36 projects selected from the advanced progressive matrix (e.g., Bouma et al., 1996; Vigneau et al., 2006). In accordance with previous research, the split-half reliability of the simplified Raven Progressive Matrices used in the current study was found to be 0.83 (Wei et al., 2012b).

### Procedure

Participants completed all seven tests on computers together in a psychological laboratory monitored by 2–4 experimenters, with each experimenter monitoring 3–5 participants. All subjects took the seven tests in a fixed order: simple subtraction, complex subtraction, figure matching, numerosity comparison, choice reaction time, nonverbal matrices reasoning, and mental rotation. Before each test, the experimenter explained the instructions presented on the computer, and then the participants completed a practice session before the formal test. Subjects’ responses were automatically recorded in a computer and sent over the Internet to a central server in the laboratory.

Each test included practice and formal test sessions. In the practice session, the message “Correct! Can you go faster?” would appear in the middle of the screen with the subjects’ correct choice, and the message “Wrong! Please try again.” would flash when subjects made an incorrect choice. There were four or six trials in the practice session. After the practice session, all participants began the formal test. Subjects were asked to respond as quickly and accurately as possible, but they were not told the specific amount of time allotted for each task. After all subjects in the psychological laboratory completed a test, they then went on to the next test.

### Data Analyses

To align with previous experiments, researchers in this study used both accuracy and reaction time (RT) as indexes for the numerosity comparison and figure matching tasks. For time-limited tasks of arithmetical computation, mental rotation, and nonverbal matrices reasoning, the Guilford correction formula was used. That is, the number of correct answers (R) minus the number of wrong answers (W) and then divided by the number of alternative answers in each trail minus one. In this study, there are two alternative answers for all tasks, so the score is s=R-W, which can reduce the impact of guessing on time-limited tasks (Guilford and Guilford, 1936). This measure has been used by many previous studies (Hedden and Yoon, 2006; Massa and Mayer, 2006; Cirino, 2011; Wei et al., 2012a,b).

For the results analyses, first, descriptive statistics was performed for all tests. Mean and SD of measures and half-split reliability for each test were calculated. Meanwhile, Pearson’s correlation coefficients among scores of all cognitive processes and arithmetical computation were calculated.

Second, a series of hierarchical regression analyses were conducted to test the role of visual perception in the correlation between numerosity processing and arithmetical computation. First, a three-step regression analysis was used to test whether numerosity processing contributed to simple subtraction and complex subtraction after controlling for age/gender differences and general cognitive processes. Then, researchers conducted a four-step regression analysis to test whether numerosity processing still contributed to simple subtraction and complex subtraction after controlling for age/gender differences, general cognitive processes, and visual perception.

Third, mediation analyses along with the bootstrapping method (Preacher and Hayes, 2008) were used to quantify the differential contributions of figure matching and numerosity processing to arithmetical performance after controlling for general cognitive processes, as well as age and gender differences.

## Results

The means and SDs of all tests are reported in Table 1. The intercorrelation coefficients of all measures for the total sample are shown in Table 2. Results showed that the arithmetical performance of adults was significantly related to a variety of cognitive processing abilities, including visual perception, numerosity processing, spatial ability, processing speed, and nonverbal matrices reasoning ability. Among these, visual figure matching has the highest correlation with simple subtraction (*r*=0.251, *p*<0.001) and complex subtraction (*r*=0.296, *p*<0.001). After controlling for age and gender differences, similar results were obtained. That means, visual perception processing was one important cognitive factor for arithmetical performance in adults.

**Table 1:** Descriptive statistics of all tasks.

Task	Index	Mean	*SD*	Split-half reliability
Simple subtraction	Adj. no. of correct trials	50.7	7.3	0.90
Complex subtraction	Adj. no. of correct trials	27.7	6.4	0.90
Numerosity comparison	Accuracy	80.7	9.1	0.88
	Reaction time	535	98	0.83
Visual figure matching	Accuracy	70.1	9.6	0.86
	Reaction time	697	297	0.81
Mental rotation	Adj. no. of correct trials	23.8	9.9	0.93
Nonverbal matrices reasoning	Adj. no. of correct trials	20.7	9.3	0.85
Choice reaction time	Reaction time	373	67	0.96

Adj. No. of correct trials=total correct trials minus total incorrect trials. This adjustment was made to control for the effect of guessing in multiple choice tests.

**Table 2:** Correlation and partial correlation after controlling for age and gender differences for all tasks.

	1	2	3	4	5	6	7	8	9
1. Simple subtraction	-	0.64^***^	0.23^***^	0.08	0.22^***^	0.16^*^	0.27^***^	0.22^***^	−0.23^***^
2. Complex subtraction	0.623^***^	-	0.22^***^	−0.05	0.27^***^	0.04	0.32^***^	0.19^***^	−0.32^***^
3. Numerosity comparison (ACC)	0.25^***^	0.24^***^	-	0.28^***^	0.37^***^	0.12	0.23^***^	0.21^***^	0.01
4. Numerosity comparison (RTs)	0.08	−0.05	0.27^***^	-	0.11	0.12	0.03	0.09	0.36^***^
5. Visual figure matching (ACC)	0.25^***^	0.30^***^	0.38^***^	0.11	-	−0.09	0.26^***^	0.31^***^	−0.06
6. Visual figure matching (RTs)	0.17^*^	0.05	0.13	0.11	−0.07	-	0.01	−0.11	−0.01
7. Mental rotation	0.25^***^	0.31	0.22^***^	0.02	0.25^***^	0.03	-	0.46^***^	−0.19^***^
8. Nonverbal matrices reasoning	0.24^***^	0.21^***^	0.23^***^	0.08	0.33^***^	−0.10	0.45^***^	-	−0.07
9. Choice reaction time	−0.19^***^	−0.29^***^	0.03	0.36^***^	−0.04	−0.01	−0.21^***^	−0.06	-

The upper portion is the partial correlation after controlling for age and gender differences; the lower part shows the full correlation among all the tasks. ACC, accuracy. Reaction times (RTs) are given in milliseconds.

* *p*<0.05; ^**^p<0.01;

*** *p*<0.001.

In addition, researchers conducted a regression analysis on simple subtraction and complex subtraction in three steps (see Table 3). Researchers found that the numerosity processing of adults is significantly correlated with simple subtraction, and independent of other general cognitive abilities, including spatial ability, processing speed, and nonverbal reasoning ability (Δ*R*^2^=0.038, *p*<0.05). The result of the complex subtraction is similar to the result of the simple subtraction (Δ*R*^2^=0.026, *p*<0.05).

**Table 3:** Results from hierarchical regression analyses for the relations of numerosity processing and arithmetic computation (simple subtraction and complex subtraction) after controlling for age/gender (setp 1), and general cognitive processes (step 2).

Predictors	Simple subtraction			Complex subtraction		
	Step 1	Step 2	Step 3	Step 1	Step 2	Step 3
	B (SE)	B (SE)	B (SE)	B (SE)	B (SE)	B (SE)
Age (month)	0.04 (0.02)^*^	0.04 (0.02)^*^	0.04 (0.01)^*^	0.03 (0.02)^*^	0.04 (0.01)^*^	0.03 (0.01)^*^
Gender	1.5 (1.02)	2.4 (1.0)^*^	2.3 (1.0)^*^	0.72 (0.90)	1.9 (0.84)^*^	1.7 (0.84)^*^
Choice reaction time	-	−0.02 (0.01)^**^	−0.03 (0.01)^**^	-	−0.03 (0.01)^***^	−0.03 (0.01)^***^
Mental rotation	-	0.13 (0.06)^*^	0.11 (0.06)^*^	-	0.16 (0.05)^**^	0.14 (0.05)^**^
Nonverbal matrices reasoning	-	0.10 (0.06)	0.07 (0.06)	-	0.04 (0.05)	0.02 (0.05)
Numerosity comparison (ACC)	-	-	0.12 (0.06)^*^	-	-	0.12 (0.05)^*^
Numerosity comparison (RTs)	-	-	0.00 (0.00)	-	-	0.00 (0.00)
	*R*^2^ =0.038^*^	Δ*R*^2^ =0.113^***^	Δ*R*^2^ =0.038^*^	*R*^2^ =0.026	Δ*R*^2^ =0.172^***^	Δ*R*^2^ =0.026^*^

Δ*R*^2^ indicates the change in the *R* Square value.

* *p*<0.05;

** *p*<0.01;

*** *p*<0.001.

Researchers used a hierarchical regression model to predict arithmetical performance from all the variables. As Table 4 shows, after controlling for other visuospatial processing skills, like figure matching, numerosity processing was no longer a significant predictor of simple subtraction (Δ*R*^2^=0.017, *p*>0.05) or complex subtraction (Δ*R*^2^=0.011, *p*>0.05). This is the most relevant result to our hypothesis, which means figure matching remained a significant predictor of arithmetical performance. To further test our hypothesis, researchers conducted another hierarchical regression model to examine whether visual perception was still relevant to arithmetical performance after controlling for gender, age, general cognitive processing, and numerosity processing. As Table 5 shows, visual perception was still associated with simple subtraction (Δ*R*^2^=0.181, *p*<0.05), but not with complex subtraction (Δ*R*^2^=0.211, *p*>0.05). These results further demonstrated the role of visual perception in arithmetic fluency.

**Table 4:** Results from hierarchical regression analyses for the relations of numerosity processing and arithmetic computation (simple subtraction and complex subtraction) after controlling for age/gender (step 1), general cognitive processes (step 2) and figure matching (step 3).

Predictors	Simple subtraction				Complex subtraction			
	Step 1	Step 2	Step 3	Step 4	Step 1	Step 2	Step 3	Step 4
	B (SE)	B (SE)	B (SE)	B (SE)	B (SE)	B (SE)	B (SE)	B (SE)
Age (month)	0.04 (0.02)^*^	0.04 (0.02)^*^	0.03 (0.02)	0.03 (0.02)	0.03 (0.02)^*^	0.04 (0.01)^*^	0.03 (0.01)^*^	0.03 (0.01)
Gender	1.5 (1.02)	2.4 (1.0)^*^	2.4 (1.0)^*^	2.33 (0.97)^*^	0.73 (0.90)	1.9 (0.84)^*^	1.7 (0.83)^*^	1.61 (0.83)
Choice reaction time	-	−0.02 (0.01)^**^	−0.02 (0.01)^**^	−0.02 (0.01)^**^	-	−0.03 (0.01)^***^	−0.03 (0.01)^***^	−0.03 (0.01)^***^
Mental rotation	-	0.13 (0.06)^*^	0.11 (0.06)^*^	0.10 (0.06)	-	0.16 (0.05)^**^	0.14 (0.05)^**^	0.13 (0.05)^**^
Nonverbal matrices reasoning	-	0.10 (0.06)	0.09 (0.06)	0.08 (0.06)	-	0.04 (0.05)	0.01 (0.05)	0.01 (0.05)
Visual figure matching (ACC)			0.12 (0.05)^*^	0.09 (0.06)			0.14 (0.05)^**^	0.11 (0.05)^*^
Visual figure matching (RTs)			0.00 (0.00)^**^	0.00 (0.00)^*^			0.00 (0.00)	0.00 (0.00)
Numerosity comparison (ACC)	-	-		0.08 (0.06)	-	-		0.08 (0.05)
Numerosity comparison (RTs)	-	-		0.00 (0.00)	-	-		0.00 (0.00)
	*R*^2^ =0.038^*^	Δ*R*^2^ =0.113^***^	Δ*R*^2^ =0.050^**^	Δ*R*^2^ =0.017	*R*^2^ =0.026	Δ*R*^2^ =0.172^***^	Δ*R*^2^ =0.037^**^	Δ*R*^2^ =0.011

Δ*R*^2^ indicates the change in the *R* Square value.

* *p*<0.05;

** *p*<0.01;

*** *p*<0.001.

**Table 5:** Results from hierarchical regression analyses for the relations of figure matching and arithmetic computation (simple subtraction and complex subtraction) after controlling for age/gender (step 1), general cognitive processes (step 2) and numerosity comparison (step 3).

Predictors	Simple subtraction				Complex subtraction			
	Step 1	Step 2	Step 3	Step 4	Step 1	Step 2	Step 3	Step 4
	B (SE)	B (SE)	B (SE)	B (SE)	B (SE)	B (SE)	B (SE)	B (SE)
Age (month)	0.04 (0.02)^*^	0.04 (0.02)^*^	0.04 (0.02)^*^	0.03 (0.02)	0.03 (0.02)^*^	0.04 (0.01)^*^	0.03 (0.01)^*^	0.03 (0.01)
Gender	1.5 (1.02)	2.40 (1.0)^*^	2.30 (1.0)^*^	2.33 (0.97)^*^	0.73 (0.90)	1.90 (0.84)^*^	1.70 (0.84)^*^	1.62 (0.83)
Choice reaction time	-	−0.02 (0.01)^**^	−0.03 (0.01)^**^	−0.02 (0.01)^**^	-	−0.03 (0.01)^***^	−0.03 (0.01)^***^	−0.03 (0.01)^***^
Mental rotation	-	0.13 (0.06)^*^	0.11 (0.06)	0.10 (0.06)	-	0.16 (0.05)^**^	0.14 (0.05)^**^	0.13 (0.05)^**^
Nonverbal matrices reasoning	-	0.10 (0.06)	0.07 (0.06)	0.08 (0.06)	-	0.04 (0.05)	0.02 (0.05)	0.01 (0.05)
Numerosity comparison (ACC)			0.12 (0.06)^*^	0.08 (0.06)			0.12 (0.05)^*^	0.08 (0.05)
Numerosity comparison (RTs)			0.01 (0.01)	0.01 (0.00)^*^			0.00 (0.01)	0.00 (0.01)
Visual figure matching (ACC)	-	-		0.09 (0.06)	-	-		0.11 (0.05)^*^
Visual figure matching (RTs)	-	-		0.00 (0.00)^*^	-	-		0.00 (0.00)
	*R*^2^ =0.038^*^	ΔR^2^ =0.129^***^	Δ*R*^2^ =0.159^*^	Δ*R*^2^ =0.181^*^	*R*^2^ =0.026	Δ*R*^2^ =0.178^***^	Δ*R*^2^ =0.197^*^	Δ*R*^2^ =0.211

Δ*R*^2^ indicates the change in the *R* Square value.

* *p*<0.05;

** *p*<0.01;

*** *p*<0.001.

**Figure 2:**
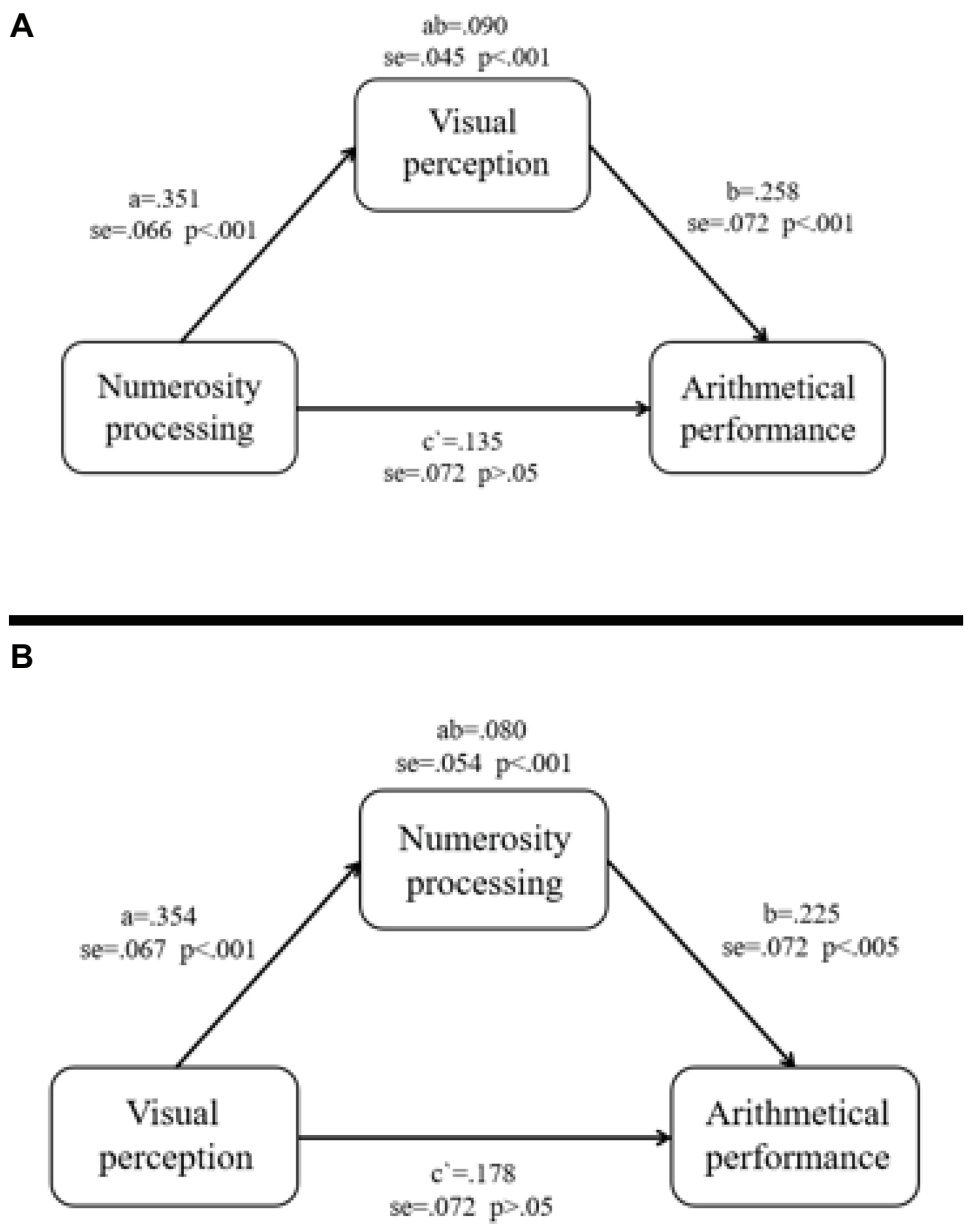
Mediation analyses for the differential contributions of numerosity processing and visual perception to arithmetical performance. **(A)** The mediation effect of visual perception on the relation between numerosity processing and arithmetical performance. **(B)** The mediation effect of numerosity processing on the relation between visual perception and arithmetical performance. (1) Arithmetical performance refers to the non-standardized residual of arithmetic computation after controlling for general cognitive processing such as non-verbal matrix reasoning, mental rotation, and choice RT, as well as age and gender differences. (2) The model is constrained by the assumption of *c*=*ab*+*c*`. *c*, direct effect of the original predictor; *ab*, indirect effect of the mediator; and *c*`, the remaining, unmediated direct effect.

To further quantify the differential contributions of numerosity comparison and visual perception to arithmetical computation, mediation analyses were conducted after controlling for gender, age, and general cognitive processing (see Figure 2). As Figure 2 shows, the arithmetical performance refers to the average scores of simple subtraction and complex subtraction. In the mediation model, the dependent variable was the residuals of arithmetical computation (SR arithmetical computation) after controlling for three general cognitive processing variables: non-verbal matrix reasoning, mental rotation, and choice RT, as well as age and gender differences. The other two variables are the standardized predicted values of numerosity comparison (accuracy and RT) and figure matching (accuracy and RT) on the SR arithmetical computation. Researchers first examined whether visual perception mediated the relation between numerosity comparison and arithmetical computation and found a full mediation (*c*=0.135, *p*>0.05), that numerosity comparison was no longer correlated with arithmetical computation (see Figure 2A). Researchers then examined whether numerosity comparison mediated the relation between visual perception and arithmetical computation. There was also a full mediation of numerosity comparison on the association, that visual perception was no longer correlated with arithmetical computation (*c`*=0.178, *p*>0.05; see Figure 2B).

## Discussion

The current study was aimed at investigating the role of visual perception in arithmetical performance of adults. Results showed that the numerosity processing of adults was significantly correlated with arithmetical performance, even after controlling for all the other general cognitive abilities including spatial ability, processing speed, and nonverbal matrices reasoning. However, after controlling for visual perception, the numerosity processing was no longer related to arithmetical performance. After controlling for numerosity processing, visual perception only correlated with simple subtraction, but not correlated with complex subtraction. Results in the current study indicate that visual perception is a critical factor for numerosity processing and arithmetical performance, it can account for the association between them.

### Relationship Between Numerosity Processing and Arithmetical Performance

The current investigation confirms that numerosity processing is significantly related to arithmetical performance in adults. These findings are similar to those of previous studies on the association between numerosity processing and arithmetical performance in children (Halberda et al., 2008, 2012; Mundy and Gilmore, 2009; Inglis et al., 2011; Libertus et al., 2011, 2013; Bonny and Lourenco, 2013).

This study revealed the correlation between numerosity processing and arithmetical performance for adults supporting the stable important role of numerical processing in mathematical performance. The current results conflict with a previous study by Inglis et al. (2011). The possible reasons might come from the different numerosity processing task and diverse mathematical performance involved in their study. Inglis et al. (2011) examined the association between numerosity processing and mathematics performance on both children and adults. They found the correlation between them only in children. It should be noted that, the task used to measure mathematical performance is the Woodcock Johnson achievement, which includes math fluency, applied problems, quantitative concepts, and number series subtests. The complex mathematical abilities tasks might affect the correlation. Actually, previous studies have shown numerosity processing correlated with arithmetical computation but not with mathematical reasoning, which measured by number series completion (Zhang et al., 2016). Except that, the different stimulus presentation time of numerosity comparison might also affect the association. In the current study, each dots array was presented for 200ms, which is the same as that used by Halberda et al. (2008). However, in the study of Inglis et al. (2011), dots array was presented for 1,500ms or presented until response.

### The Role of Visual Perception in the Arithmetical Performance of Adults

The main contribution of the current study is the examination of the visual perception hypothesis on adults. This actually tested the developmental cognitive mechanism of arithmetical performance. Researchers found that visual perception is a common processing mechanism of numerosity processing and arithmetical performance, which is similar to previous studies that focused on children. As expected, researchers found that visual perception, measured by a figure matching test, can fully account for the correlation between numerosity processing and arithmetical performance, even after controlling for other general cognitive processes.

The correlation of visual form perception with numerosity processing and arithmetic processing might first come from the form perception they shared. Both numerosity processing and arithmetical performance relied on visual form perception. For numerosity processing, dot layouts other than a single dot could be considered a type of form. The visual characteristics of a numerosity array are defined by the structural relationships among its elements. Szwed et al. (2009) showed that the vertices of lines were an invariant visual feature of line drawings of objects and symbols. The dots in a dot array play the same role as the vertices in these graphs. Therefore, in the process of digital judgment, the perception of the structural relationship between points is the key to the number extraction. Actually, numerosity comparison was heavily affected by visual properties including total surface area, envelope area, item size, circumstance, and density (Gebuis and Reynvoet, 2012). Arithmetic computation is typically based on the rapid perception of Arabic digits, which is also a type of visual form.

Similar results in adults also revealed the importance of fluency processing in the correlation between numerosity processing and arithmetical performance. That means, visual form perception is the underlying cognitive factor of numerosity processing and arithmetical performance. That might come from the shared processing mechanism of fluency processing on the visual form. Adults can extract the arithmetical facts accurately and fast. In our study, both figure matching and numerosity comparison tasks involve the short presentation of stimuli, so the speed of visual perception may be the key. Participants need to quickly encode sensory input, obtain information from long-term memory, and integrate different information into working memory. Actually, previous studies showed that the quick processing during numerosity comparison, or those with a stimulus presentation time of less than 300ms, correlated with arithmetic fluency (Halberda et al., 2008, 2012; Gebuis and Reynvoet, 2012; Lourenco et al., 2012; Wei et al., 2012a). When the presentation time was longer than 300ms, some studies found the correlation (Libertus et al., 2013; Keller and Libertus, 2015; Matthews et al., 2016), but others did not (Price et al., 2012; Fuhs and Mcneil, 2013; Kolkman et al., 2013; Sasanguie et al., 2014).

### Implications, Limitations, and Future Studies

The finding that differences in visual perception affect the association between numerosity processing and arithmetical performance supports the visual perception hypothesis, which provides important inspiration for mathematics education. According to the previous studies, the important role of visual form perception in the association between numerosity processing and arithmetical performance is not only presented in children, but also supported by adults. The visual perception hypothesis is stable for both children and adults. This conclusion allows us to do in-depth research on the impact of visual perception on various fields in the future.

This study was not without limitations. The current study did not directly control visuospatial attention. Visuospatial attention always contains visual perception. For example, the Visual Form Discrimination Test is a complex multiple-choice, matching-to-sample task of visual attention. During the attention processing portion, participants needed to complete the tasks with visual forms. In the current study, although figure matching is a typical visual perception task (Ekstrom et al., 1976; Zhou et al., 2015; Zhang et al., 2019), this task might rely on attention resources. Future studies need to directly control for visuospatial attention. Visuospatial attention can be measured with IVA (Chen et al., 2003) or AUT program (Bernhofs et al., 2015).

## Conclusion

The results of the current study indicated that visual perception correlated with numerosity processing and arithmetical performance and was the shared processing mechanism of both of them for adults. All of these results supported and confirmed the stability of the visual perception hypothesis, which states that visual perception underlies both numerosity processing and arithmetical computation from childhood to adulthood.

## Data Availability Statement

The raw data supporting the conclusions of this article will be made available by the authors, without undue reservation.

## Ethics Statement

The studies involving human participants were reviewed and approved by Beijing Normal University. Written informed consent to participate in this study was provided by the participants’ legal guardian/next of kin.

## Author Contributions

XH was responsible for the operation, data analysis, and writing of the experiment. YZ was responsible for the operation of the experiment and the analysis of part of the data. XZ was responsible for the operation of the experiment and the proofreading of the article. JZ was responsible for later period data analysis and article revision. All authors contributed to the article and approved the submitted version.

## Funding

This research was supported by three grants from the Natural Science Foundation of China (Nos. 31700971, 31221003, and 31271187).

## Conflict of Interest

The authors declare that the research was conducted in the absence of any commercial or financial relationships that could be construed as a potential conflict of interest.

## Publisher’s Note

All claims expressed in this article are solely those of the authors and do not necessarily represent those of their affiliated organizations, or those of the publisher, the editors and the reviewers. Any product that may be evaluated in this article, or claim that may be made by its manufacturer, is not guaranteed or endorsed by the publisher.

## Footnotes

^1^ http://www.dweipsy.com/lattice/#/home
